# Supplementary material for: Integrative Analysis of Metabolome and Transcriptome of Carotenoid Biosynthesis Reveals the Mechanism of Fruit Color Change in Tomato (Solanum lycopersicum)
Source: Int J Mol Sci. 2024 Jun 12;25(12):6493. doi: 10.3390/ijms25126493 (PMC11204166; doi:10.3390/ijms25126493)
Supplement: Supplementary file 1 [file ijms-25-06493-s001.zip › Figure S1.pdf]

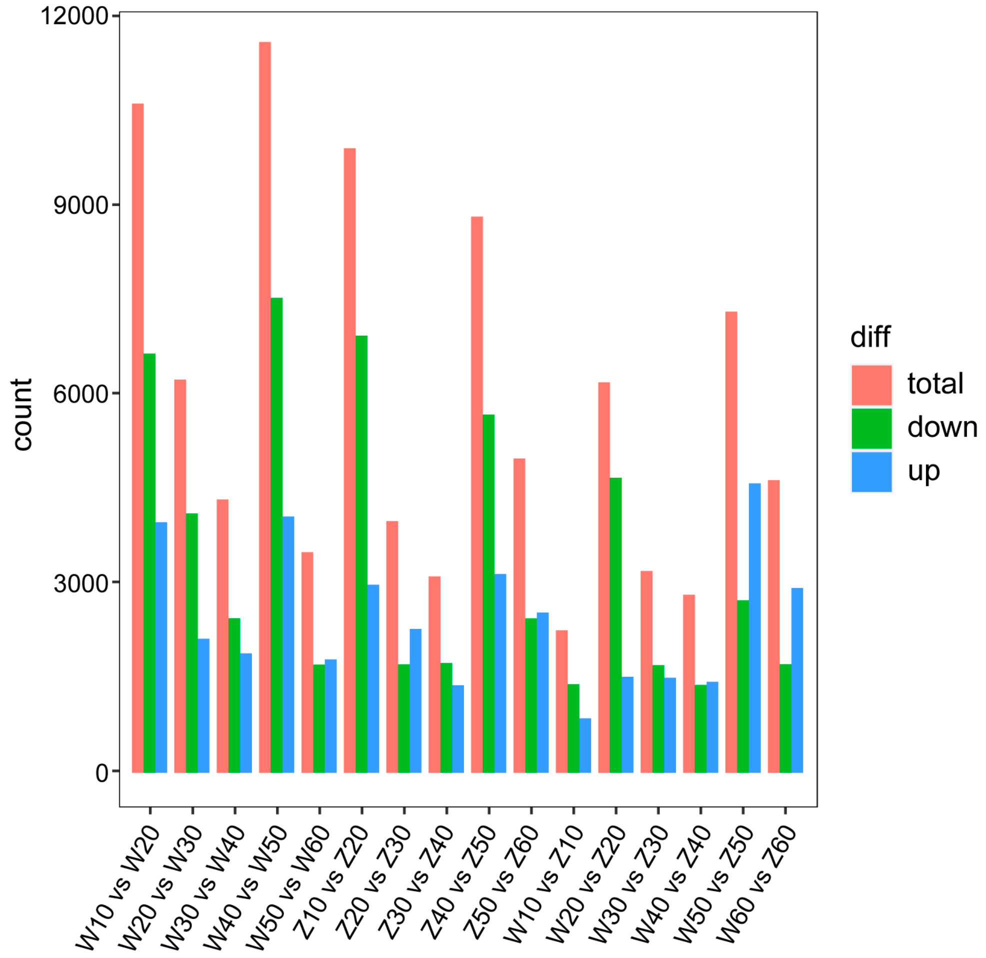

Figure S1. Statistics of differently expressed genes between different developmental periods of tomato fruits between two varieties.
