# Supplementary material for: Integrative Analysis of Metabolome and Transcriptome of Carotenoid Biosynthesis Reveals the Mechanism of Fruit Color Change in Tomato (Solanum lycopersicum)
Source: Int J Mol Sci. 2024 Jun 12;25(12):6493. doi: 10.3390/ijms25126493 (PMC11204166; doi:10.3390/ijms25126493)
Supplement: Supplementary file 1 [file ijms-25-06493-s001.zip › Figure S4.pdf]

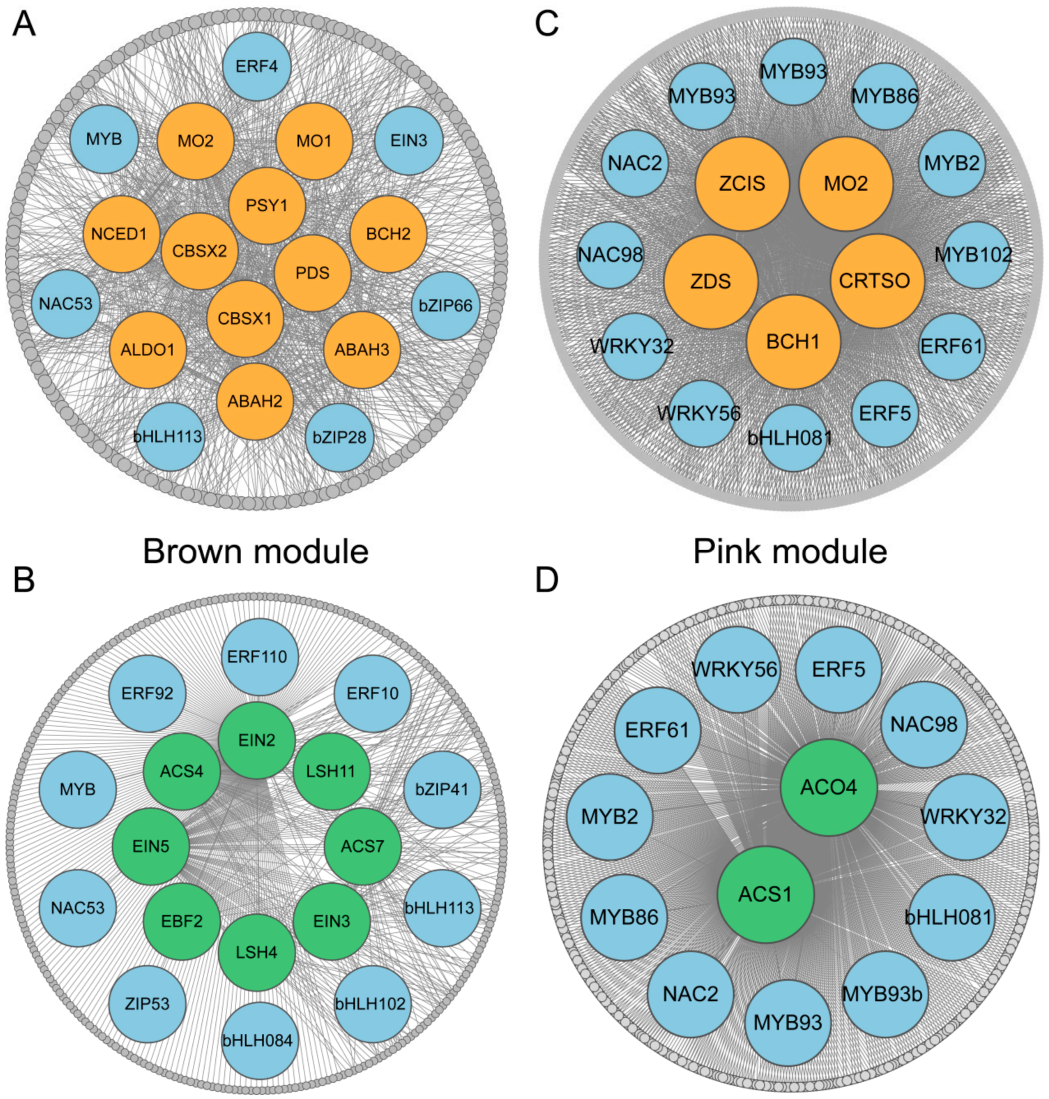

Figure S4. The correlation network in modules highly related to carotenoid and ethylene biosynthesis and signal transduction. A, B: the correlation network in brown module; C, D: the correlation network in pink module. The transparency of the edges indicates the weight value between two genes.
