# Supplementary figures and images for: Integrative Analysis of Metabolome and Transcriptome of Carotenoid Biosynthesis Reveals the Mechanism of Fruit Color Change in Tomato (Solanum lycopersicum)
Source: Int J Mol Sci. 2024 Jun 12;25(12):6493. doi: 10.3390/ijms25126493 (PMC11204166; doi:10.3390/ijms25126493)

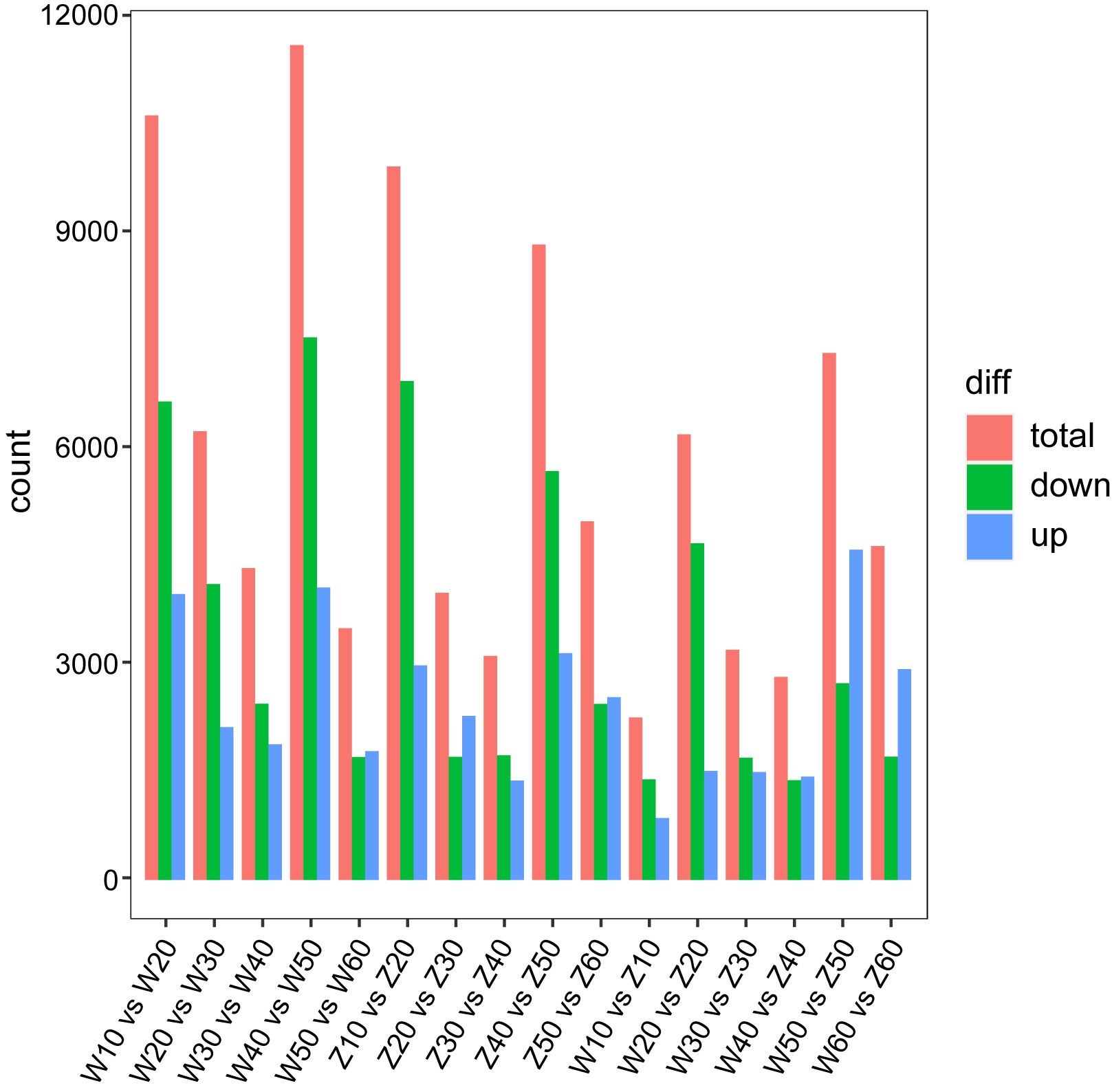

Supplement: Supplementary file 1 [file ijms-25-06493-s001.zip › Figure S1.tif]

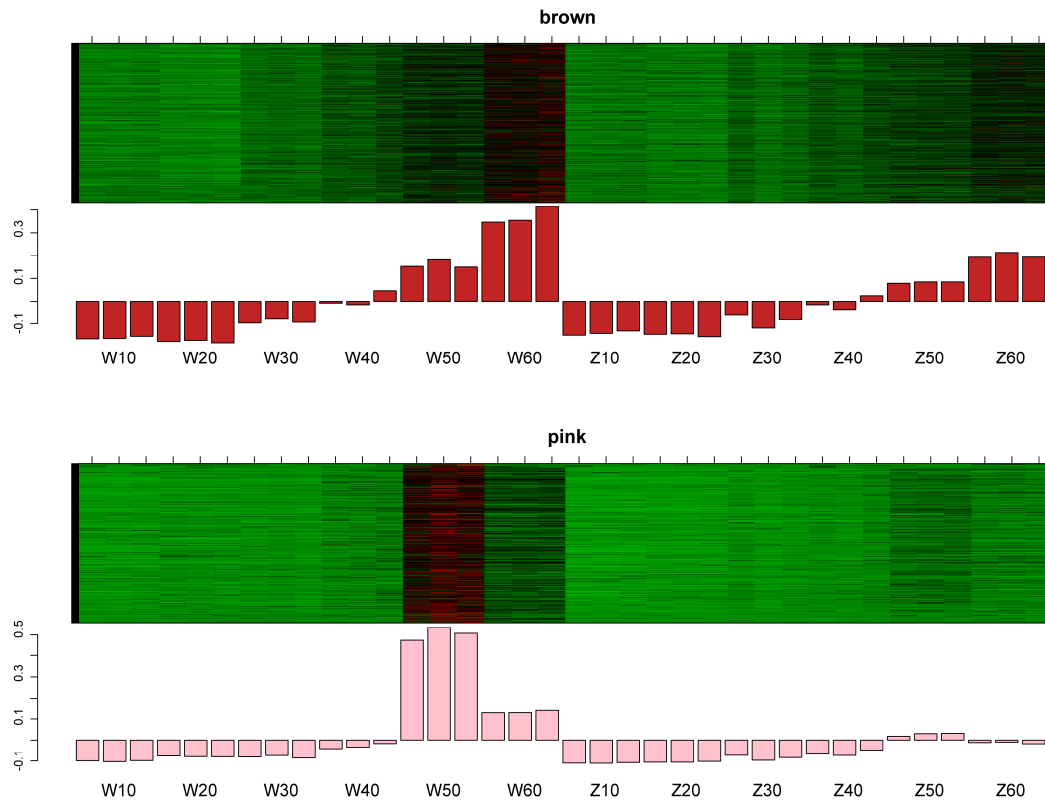

**Figure S2.** Heat-map of expression patterns of genes from brown and pink modules.

Supplement: Supplementary file 1 [file ijms-25-06493-s001.zip › Figure S2.pdf]

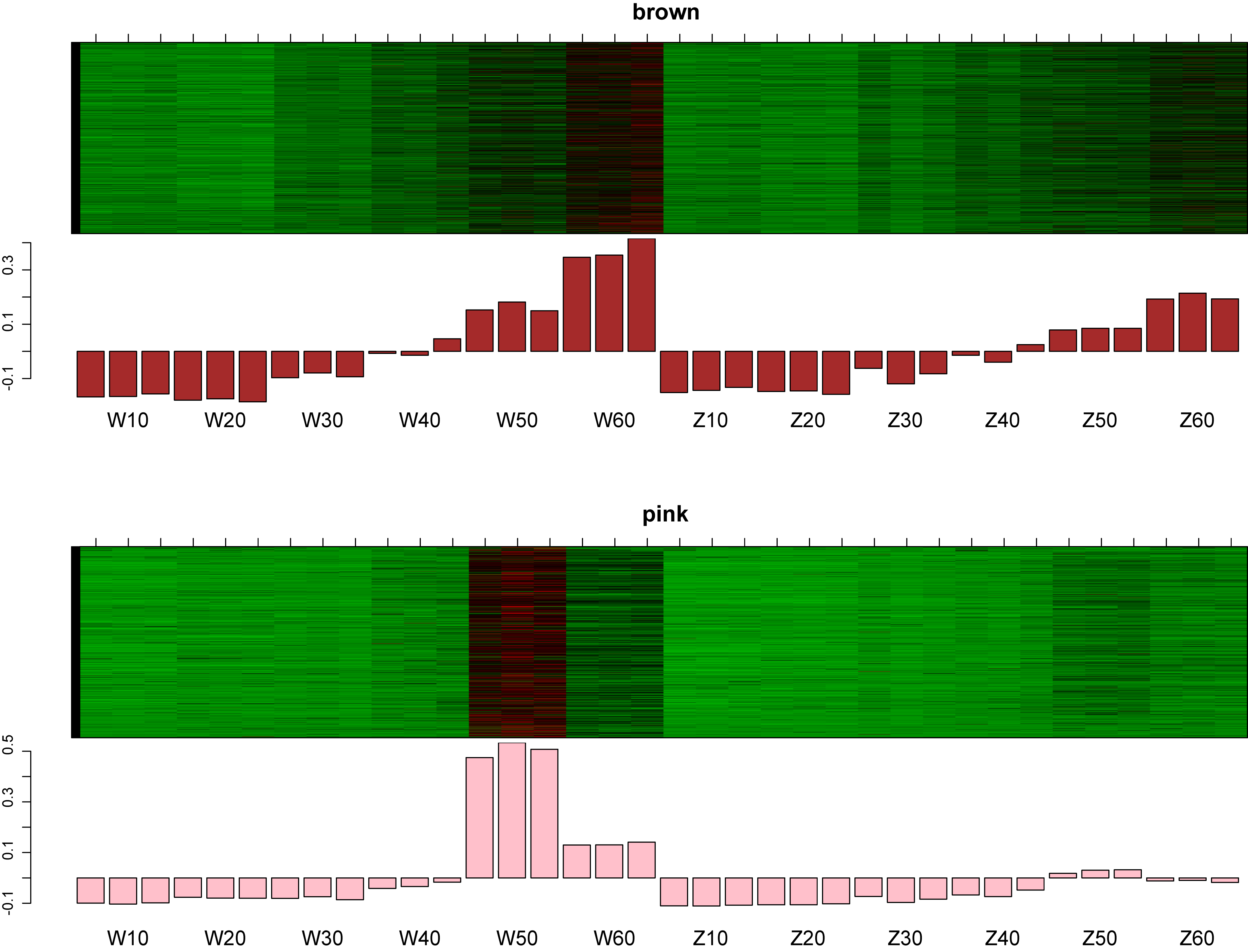

Supplement: Supplementary file 1 [file ijms-25-06493-s001.zip › Figure S2.tif]

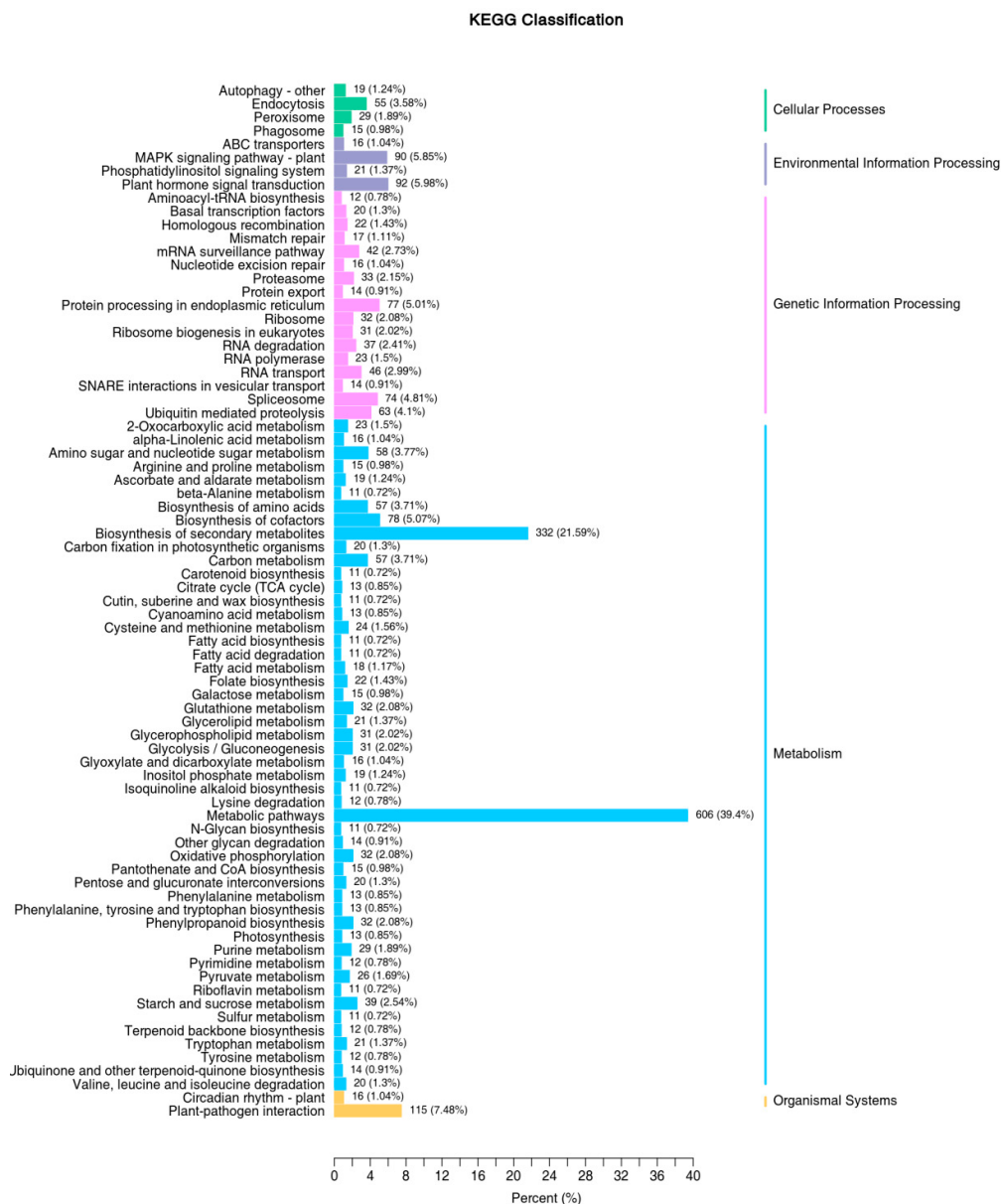

**Figure S3.** KEGG pathway-classification analysis of brown module.

Supplement: Supplementary file 1 [file ijms-25-06493-s001.zip › Figure S3.pdf]

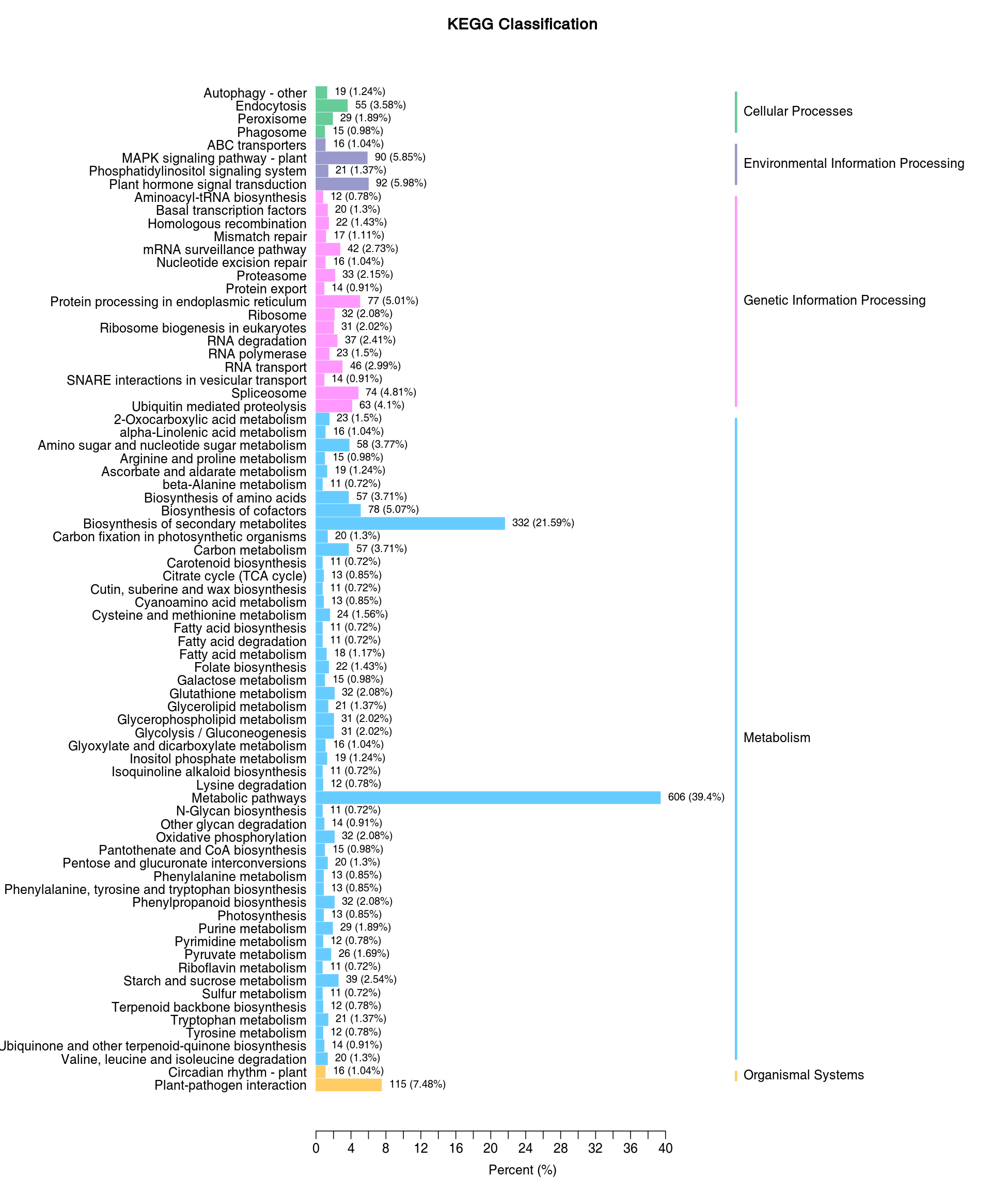

Supplement: Supplementary file 1 [file ijms-25-06493-s001.zip › Figure S3.png]

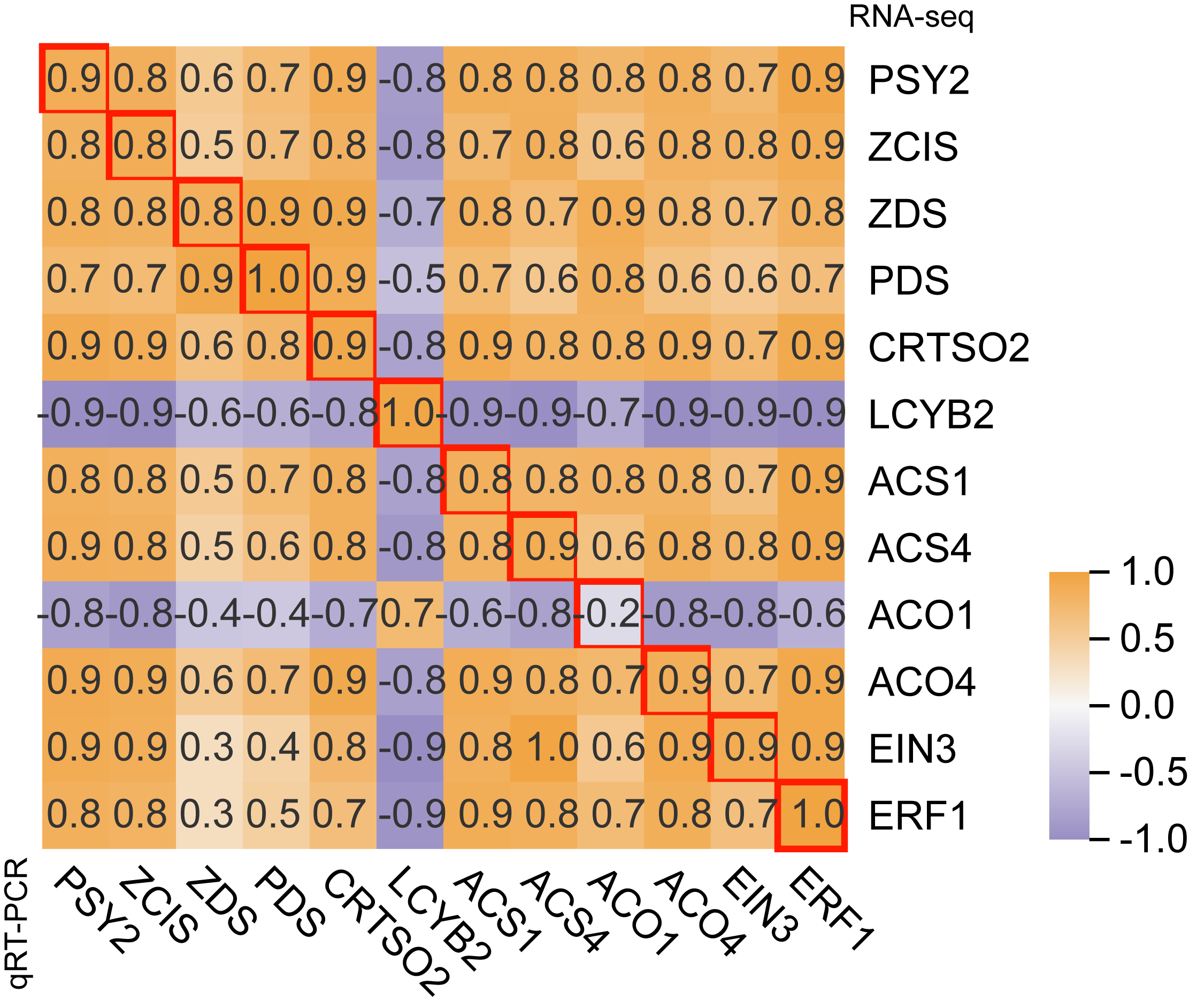

Supplement: Supplementary file 1 [file ijms-25-06493-s001.zip › Figure S5.tif]
